# Use of Laser-Induced Breakdown Spectroscopy for the Detection of Glycemic Elements in Indian Medicinal Plants

**DOI:** 10.1155/2013/406365

**Published:** 2013-10-21

**Authors:** Prashant Kumar Rai, Amrita Kumari Srivastava, Bechan Sharma, Preeti Dhar, Ajay Kumar Mishra, Geeta Watal

**Affiliations:** ^1^Department of NMR, All India Institute of Medical Sciences, New Delhi 110029, India; ^2^UJ Nanomaterials Science Research Group, University of Johannesburg, P.O. Box 17011, Doornfontein, Johannesburg 2028, South Africa; ^3^Alternative Therapeutics Unit, Drug Discovery & Development Division, Medicinal Research Lab, Department of Chemistry, University of Allahabad, Allahabad 211002, India; ^4^Department of Biochemistry, University of Allahabad, Allahabad 211002, India; ^5^Department of Chemistry, State University of New York, 1 Hawk Drive, New Paltz, NY 12561, USA

## Abstract

The demand for interdisciplinary research is increasing in the new millennium to help us understand complex problems and find solutions by integrating the knowledge from different disciplines. The present review is an excellent example of this and shows how unique combination of physics, chemistry, and biological techniques can be used for the evaluation of Indian medicinal herbs used for treating diabetes mellitus. Laser-induced breakdown spectroscopy (LIBS) is a sensitive optical technique that is widely used for its simplicity and versatility. This review presents the most recent application of LIBS for detection of glycemic elements in medicinal plants. The characteristics of matrices, object analysis, use of laser system, and analytical performances with respect to Indian herbs are discussed.

## 1. Introduction

The World Health Organization estimates that about 80% of the world's population relies on herbal medicines for primary healthcare [1, 2]. Knowledge of herbal medicines evolved by trial and error and was passed orally from generation to generation [3, 4]. It is only during the last five decades that ethnobotanical research has documented numerous medicinal plants that were earlier not known to the scientific world [5–9]. This body of research has been possible in part due to the availability of several analytical techniques and isolation processes for studying the phytoconstituents of plants. 

One important analytical technique that has led to advances in natural product research is laser induced breakdown spectroscopy (LIBS). In this paper, we review the applications of this technique and then review its use for the evaluation of Indian medicinal plants used for treating diabetes mellitus. Specifically, we review what these glycemic elements are and how LIBS has been used to detect these elements in plants known to have hypoglycemic property.

A number of plant species have hypoglycemic property [10, 11]. Despite the availability of many antidiabetic medicines, screening for antidiabetic drugs from natural sources is an attractive proposition. First, natural medicines are widely used in many developing and underdeveloped countries. Second, natural medicines are believed to have minimal side effects and are therefore preferred over synthetic medicines.

## 2. Laser-Induced Breakdown Spectroscopy (LIBS)

The LIBS technique is used for qualitative and quantitative analysis of trace elements. It was used for the first time in 2008 to study the presence of certain trace elements in medicinal plants with hypoglycemic effects [12]. This technique is used to analyze the spectral emission from laser-induced plasmas, the plasma emission intensity being proportional to the abundance of an element in the sample. The relative simplicity and capability of fast multielemental analyses of solid, liquid, or gaseous samples makes LIBS an ideal tool to study a wide range of samples. These include metallurgical and solid samples, colloidal and liquid samples, particles, and gases. While the qualitative analysis of a sample is straightforward, the quantitative results of elemental compositions from LIBS measurements require much more effort. Although the use of LIBS has been most popular in metallurgical samples, in recent years, it has been used to study environmental and biological samples, advanced materials such as semiconductors, for online sample analysis, for remote analysis of nuclear power stations, and for depth profiling of a field. 

### 2.1. LIBS Applications

#### 2.1.1. Liquids

Initially, liquid analysis with LIBS was not popular because of problems such as sloshing, splashing, and focal length changes with a high repetition rate laser. Apart from this, high local density within the liquid caused intrinsic complexity with LIBS analysis; the spectral transition in comparison to that for a rarefied gas, considerably broadens when the high collision rate within the plasma is confined in a liquid. If attributes like minimal sample preparation and high detection sensitivity are resolved, then LIBS offers great potential for detection of elements in liquids. Consequently, various attempts were made by several researchers to overcome the problems encountered for liquid samples [13–15].

#### 2.1.2. Aerosols and Gases

In today's world, *in situ* and real time techniques have a range of applications in the analysis of small particles that range from submicrometer to several micrometers in diameter. Such techniques are useful in atmospheric sciences, process monitoring and control, and effluent waste stream monitoring. The LIBS technique was used to detect chromium in aerosols using a constant-output aerosol generator called atomizer [16]. In multiphoton ionization, the dominant mechanism for plasma formation is at 266 nm and avalanche ionization at 1064 nm, while both are almost equal contributors at 532 nm. The results show that, to have a maximum efficiency of ionization, an increase of laser energy/pulse is needed. Once the maximum efficiency for ionization is reached, no further increase in incident energy is necessary. For calcium and magnesium based aerosols, LIBS was used for quantitative analysis of size, mass, and composition of individual micron- to submicron-sized aerosol particles over a range of characterized experimental conditions [17].

#### 2.1.3. Metallurgical Samples

 LIBS has been used for direct and rapid determination of various types of trace metals. For analyzing the various trace elements, such as Mg, Cu, Cr, Si, and Ca, in rock samples, a time-resolved LIBS (TRELIBS) technique was used by Song et al. [18]. The analytical signal of trace elements was integrated within 20 n sec after an optimal gate delay time of 200 n sec. The detection limit (S/N ratio = 3) was in the order of 5–100 parts per million (ppm). Precision was typically 5–10% relative standard deviation (RSD). This methodology was used to determine several elements (Al, Cu, Fe, Pb, and Sn) in solid zinc alloys [19].

#### 2.1.4. Environmental Samples

With the increased research awareness, LIBS has also been used for environmental monitoring, specifically for online and remote analysis of potential hazards. The United States Environmental Protection Agency (EPA) encourages facilities to evaluate the use of real-time emissions monitoring technology in industry. Theriault et al. used LIBS for screening heavy-metal-contaminated soils using an *in situ* probe [20]. The detection limits can meet the EPAs site screening levels (SSLs) for several key metal contaminants in sand, although the probe response is affected by the soil matrix conditions like rain amount and water content. Knight et al. used LIBS for stand-off analysis of soil at reduced air pressure and in a simulated Martian atmosphere (5–7 Torr pressure of CO_2_) showing the feasibility and scope of the use of LIBS in space exploration [21]. However, the extent to which the method can provide quantitative information in space remains to be seen and must be thoroughly studied before the technique is deployed.

#### 2.1.5. Nonmetallic Solids

LIBS was adapted in mining and coal industries for both exploration as well as ore body imaging; it is employed by using an optical fiber bundle with wide acceptance angle, placed at a distance to find out the elemental content of the mineral core drill sample. LIBS was also used in the study of archaeological objects and for conservation and restoration of cultural heritage, though the main focus has been on laser artwork cleaning. Mineral assaying applications were performed using TRELIBS as reported by Bolger [22]. They used a Q-switched Nd:YAG laser to test lengths of drill core, with remote LIBS signal acquisition via a bare optical fiber bundle coupled to a spectrometer. High linear correlations (*R* ^2^ > 0.92) were obtained for Cr, Cu, Mn, Ni, and Fe, which appeared in concentration range of 200 ppm but were actually 10% of 200 ppm, as compared to the normalized atomic emission intensities of these elements in the laboratory result. The detection limits for these and other elements were extrapolated to be around 300 ppm. Fabre et al. used LIBS in geological materials for the determination of lithium in melt inclusions, quartz, and associated fluid inclusions and then compared the results (obtained with LIBS) with the result of electron microprobe technique. The results obtained with LIBS were in good agreement with bulk and microprobe data obtained for the same minerals [23].

#### 2.1.6. Plant Materials

In recent years, LIBS has received much attention with applications to solid and liquid analysis of plants materials [24–30]. Due to shock wave generation and splashing phenomenon, repetition rate becomes an important parameter when analyzing a sample in the liquid phase. It has been observed that the average spectra is preferred instead of single-shot spectrum for enhancement of the signal-to-background and signal-to-noise ratio and to get reproducibility [15]. The essential challenge in LIBS spectroscopy is the calibration. Classical approach is the use of an internal standard of known or constant concentration [31–33]. 

#### 2.1.7. Advanced Materials

In the field of surface analysis, including composition mapping and in-depth profile analysis of advanced materials, the most advanced LIBS techniques have been used. Major progress at the nanometric range has been achieved for depth-resolved measurement. Detailed spatial information by imaging mode of LIBS has been achieved by point-to-point mapping using a spherical lens focusing system, when a multidimensional detector is used. Romero and Laserna used multichannel LIBS to generate selective chemical images for Ag, Ti, and C from silicon photovoltaic cells [34]. Both surface and depth distributions were amenable with the help of this approach. Lateral resolution of 80 nm and depth resolution of better than 13 nm for TiO_2_ coatings were achieved. The surface analyses of photonic-grade silicon were also tested using LIBS technique [35]. A total area of 3–2.1 mm^2^ was analyzed with a lateral resolution of 70 mm and depth resolution of about 0.16 mm. Two-dimensional (2D) and three-dimensional (3D) distribution maps of carbon contamination on silicon were presented. The use of LIBS for quantitatively mapping the multielement distribution on polish rock and for copper in printed circuits was proposed by Yoon et al. and Kim et al. [36, 37].

#### 2.1.8. Miscellaneous Applications

LIBS application for the online multielement analyses of glass melts in a vitrification process of high-level liquid waste (HLLW) was investigated by Yun et al. [38]. Twelve different HLLW glass melts with a complex composition of about 27 chemical elements were simulated on a laboratory scale, varying the HLLW component concentration. The analytical method was calibrated by real-time analyses of the reference glass at 1200°C. The LIBS results were also well presented, and comparisons were done with those determined by XRF and ICP-AES. The influence of the matrix on the LIBS of magnesium was presented by Gornushkin et al. and Rai et al. [39, 40]. The surface density normalization method works well for the reduction of the matrix effect in the determination of Mg in powdered samples of different bulk compositions. According to Kurniawan et al. [41], the relative error of 10% and a precision of 10–20% were obtained for the determination of Mg in several certified samples. 

## 3. Diabetes, Natural Products, and LIBS

### 3.1. Diabetes Mellitus and Its Incidence

Diabetes mellitus, hereafter referred to as diabetes, is a heterogeneous disorder characterized by excess blood glucose due to improper metabolism of proteins, fats, and carbohydrates. Diabetes is characterized by elevated fasting blood glucose (FBG) and postprandial glucose (PPG) levels. The body is unable to utilize available blood sugar (glucose), either due to unavailability of insulin caused by inactivation of *β*-cells of pancreas or due to improper utilization of insulin (also known as insulin resistance) due to insensitiveness of cell receptors to insulin [42].

The World Health Organization (WHO) has listed diabetes as one of the major killers of our time. About 225 million people worldwide are estimated to be suffering from diabetes. This number may probably double by the year 2030. It is the 7th leading cause of death even in a developed country like the US [43]. India has been labeled as “Diabetes capital of the world” due to highest incidence of diabetes. Each day, more than 2,200 people are diagnosed with diabetes. Unfortunately, more than 50% of diabetics are not aware of their condition. 

### 3.2. Pathogenesis of Diabetes

**Figure 1:**
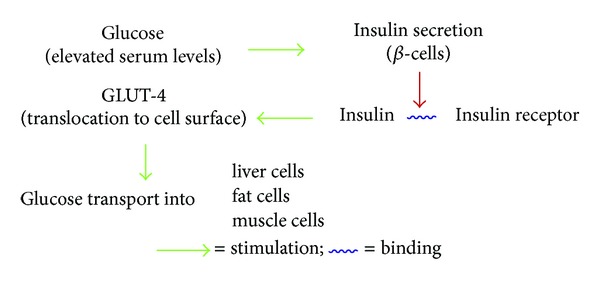
Mechanism of glucose delivery.

Insulin is secreted, in response to elevated serum glucose levels, by *β*-cells of the islets of Langerhans in the pancreas. The *α*-cells of islets of Langerhans secrete glucagon, a hormone with action nearly opposite of insulin. The role of insulin is to stimulate the GLUT-4 glucose transporter. GLUT-4 is the most important of the glucose transporter molecules, and its insertion into the muscles and adipose cell membranes serves to facilitate glucose delivery into these cells. This is the only mechanism by which the glucose can be delivered to fat, muscle, and also liver cells (Figure 1). Failure at any step of the above-mentioned mechanism of glucose delivery triggers diabetes. 

### 3.3. Types of Diabetes

#### 3.3.1. Type 1

Formerly known as insulin-dependent diabetes mellitus (IDDM), it occurs when the *β*-cells of the pancreatic islets of Langerhans are destroyed, such that insulin production is grossly impaired. Thus, type 1 diabetes is invariably treated with insulin. It is also referred to as juvenile diabetes.

#### 3.3.2. Type 2

Formerly known as noninsulin-dependent diabetes mellitus (NIDDM), typical type 2 diabetes is rarely found as an isolated abnormality. Obesity, hypertension, dyslipidemia, and hyperurinemia appear to cluster in the same individuals. Insulin resistance may be the underlying cause of this type of metabolic syndrome. Typical dyslipidemia associated with type 2 diabetes is hypertriglyceridemia and hypocholesterolemia, [44–47]. The mechanism by which hyperinsulinemia can lead to hypertension and hypertriglyceridemia has already been proposed [48–53]. 

#### 3.3.3. Type 3

The term “type 3 diabetes” reflects the fact that Alzheimer's disease (AD) represents a form of diabetes that selectively involves the brain and has molecular and biochemical features that overlap with both type 1 and type 2 diabetes mellitus. The characteristic molecular and biochemical abnormalities associated with AD include cell loss, increased activation of prodeath genes and signaling pathways, impaired carbohydrate metabolism, chronic oxidative stress, DNA damage, and so forth. Currently, there is a rapid growth in the literature pointing toward insulin deficiency and insulin resistance as mediators of AD-type neurodegeneration, but this surge of new information is riddled with conflicting and unresolved concepts regarding the potential contributions of type 2 diabetes mellitus and obesity to AD pathogenesis [54]. However, extensive disturbances in brain insulin signaling could account for the majority of molecular and biochemical lesions in AD. It has been reported that type 2 diabetes mellitus causes brain insulin resistance and oxidative stress. Experimental brain diabetes produced by intracerebral administration of streptozotocin (STZ) shares many features with AD and is treatable with insulin sensitizer agents, that is, drugs currently used to treat type 2 diabetes mellitus (T2DM) [54].

## 4. Indian Plants and Their Glycemic Elements

Several Indian plants and their glycemic elements have been discussed in the literature.

### 4.1. *Cynodon dactylon* (Family: Poaceae)

Commonly known as “Dhoob” in India, is a weed, and is regarded to possess antidiabetic properties. Aqueous and ethanolic extracts of whole plant were found to have hypoglycemic and hypolipidemic effects in STZ-induced diabetic rats at a dose of 500 mg kg^−1^ [55, 56]. Recently, antioxidant activity and glycemic elemental study have been reported on the aqueous extract of the leaves of this plant [24, 25]. Results of the LIBS analysis of *C. dactylon* indicate that its extracts consist of elements like Mg, C, and Ca in the spectral range 200–500 nm. 

Because the intensity of a spectral line of a particular element present is directly proportional to its concentration, the proportion of the concentration of these elements could be evaluated by taking the ratio of intensity of these elements and the intensity of a reference line. In the aforesaid study, C III (229.4 nm) is the reference as carbon (C) being an essential constituent of trace elements present in plants, is abundant. C III (229.4 nm) line is interference-free line and, hence, fulfills the requirement to be chosen as a reference line. The intensity ratio of Mg/C and Ca/C has been calculated and tabulated in Table 1.

### 4.2. *Emblica officinalis* (Family: Euphorbiaceae)

Commonly known as “Amla” in Hindi, grows in India as well as tropical and subtropical regions of the world. The fruit of *E. officinalis *has been reported to have potent antimicrobial, antioxidant, hepatoprotective, antitumor, and hypolipidemic properties [57, 58]. The aqueous extract of the *E. officinalis* seeds has antidiabetic and antioxidant activities [59, 60]. The LIBS spectrum of *E. officinalis* fruit extract was taken in different spectral ranges at optimized experimental conditions. It revealed the presence of Mg, Na, Cl, Ca, H, O, C, and N elements in the spectral range of *λ* 200–900 nm. According to the Boltzmann distribution law, intensity is directly related to concentration [61], therefore, the intensity of observed spectral lines corresponding to the major and minor elements present in the extract indicates their concentrations and helps define their role in STZ-induced diabetes and its stress management [12, 24, 25, 55–60].

### 4.3. *Ficus bengalensis* (Family: Moraceae)

**Scheme 1:**
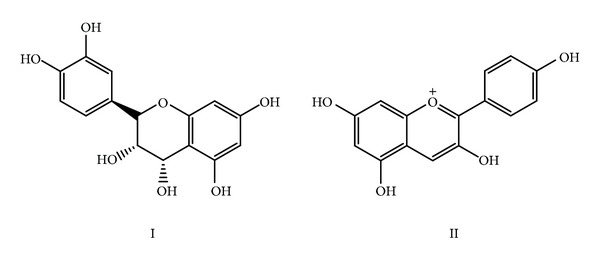
Leucocyanidin (I) and pelargonidin (II) isolated from the bark of *Ficus bengalensis* show hypoglycemic activity.

Commonly known as “Indian Banyan Tree or Bur,” is distributed throughout India. A glycoside called bengalenoside was isolated from the bark and showed more potent hypoglycemic action compared to the crude ethanolic bark extract, and the activity being was half that of the synthetic drug tolbutamide [62]. Oral administration of bark extract showed significant antihyperglycemic effect in STZ diabetic rats by raising serum insulin levels. Leucocyanidin and pelargonidin compounds (Scheme 1) isolated from the bark have also shown hypoglycemic activity [63–65]. Most recently, the hypoglycemic as well as antidiabetic properties have been reported in aerial roots of this tree [66]. The LIBS results showed a higher concentration of Mg and Ca in aqueous extract of *Ficus bengalensis *as compared to other elements present.

### 4.4. *Moringa oleifera* (Family Moringaceae)

Commonly known as Drumstick tree, is indigenous to Northwest India. Most parts of this tree possess antimicrobial activity [67]. The tree is well known for its pharmacological actions and is used for the traditional treatment of diabetes mellitus, hepatotoxicity [68], rheumatism, venomous bites, and cardiac stimulation. The leaves of *M. oleifera* are lopped for fodder [69] and have been used as antiulcer, diuretic, antiinflammatory and for wound healing [67, 70]. Ethanolic extract of leaves has shown antifungal activity against a number of dermatophytes [71, 72]. The aqueous extract of the leaves has been found to possess antidiabetic activity [73]. The LIBS spectrum of *M. oleifera *fruit extract was taken at optimized experimental conditions. It revealed the presence of Mg, Ca, H, O, C, and N elements in the spectral range of 200 nm to 400 nm. The proportion of the concentration of these elements could be evaluated by taking the ratio of intensity of these elements with intensity of reference lines (C and O, respectively, as they are essential elements of plant materials and hence are in abundance).

### 4.5. *Momordica charantia* (Family: Cucurbitaceae)

Commonly known as bitter gourd (melon) or Karela, is widely planted in tropical areas and is usually consumed as a vegetable. Bitter gourd has also been frequently used as a medicinal herb in Asia, Africa, and South America because of its antidiabetic, anthelmintic, abortifacient, antibacterial, antiviral, and chemopreventive functions [74, 75]. In addition, freeze-dried *M. charantia* powder was found to have antidiabetic activity [26]. LIBS spectrum of the freeze-dried *M. charantia* fruit powder was recorded to identify its glycemic trace elements that may be responsible for diabetes management in biological systems. The LIBS results point out that dried fruit pellet of *M. charantia *consists of elements like Na, K, Mg, Ca, Fe and Al in the spectral range of 200 nm–500 nm. The concentrations of these elements were determined by calibration-free (CF)-LIBS method. This approach requires extensive data processing and, hence, a data processing algorithm was developed [76] to quantify the effect of different CF parameters using a code in MATLAB [26]. 

### 4.6. *Psidium guajava* (Ripe Fruit Peel): (Family: Myrtaceae)

Is a semideciduous tropical tree commonly known as “Amrood” in Hindi and is widely grown throughout India for its fruit. A high percentage of vitamin C, carotene, vitamins B_1_, B_2_, and B_6_, and free sugars (glucose, fructose, and sucrose) have been reported in this fruit [77]. The *P. guajava* fruit has been discovered as a new source of antioxidants and is reported to have hypoglycemic effect [78] on blood glucose level (BGL) of normal and STZ-induced mild as well as severely diabetic rats during glucose tolerance test (GTT) and PPG studies, respectively. Surprisingly, aqueous extract of the ripe fruit peel was discovered to be hyperglycemic [79], and the observed hyperglycemic effect has been correlated to its low Mg concentration [27]. 

### 4.7. *Trichosanthes dioica* Roxb. (Family: Cucurbitaceae)

It is a dioecious perennial herbaceous vegetable. It is commonly known as “Parval” in Hindi and is widely grown throughout India. Its fruits are a rich source of vitamin C and minerals (Mg, Na, K, Cu, and S). Normal animals fed with a diet consisting of shade dried fruits of *Trichosanthes dioica*, have shown hypocholesterolemic, hypotriglyceridemic, and hypophospholipedemic effects [80]. Normal and diabetic human volunteers given the direct intake of fruit and pulp also have shown hypocholesterolemic and hypotriglyceridemic effects [81]. Direct feeding of the seeds of this fruit was also found to be effective in lowering the serum lipid profile of normal and mild diabetic human subjects [82] and albino rabbits [83]. Effect of fruit powder has been studied on blood sugar and lipid profile of normal albino rabbits [84]. Aqueous extract of seeds and leaves have glycemic elemental profile and have shown antihyperglycemic, hepatoprotective properties [28–30, 85–88].

### 4.8. *Withania coagulans* (Family: Solanaceae)

**Scheme 2:**
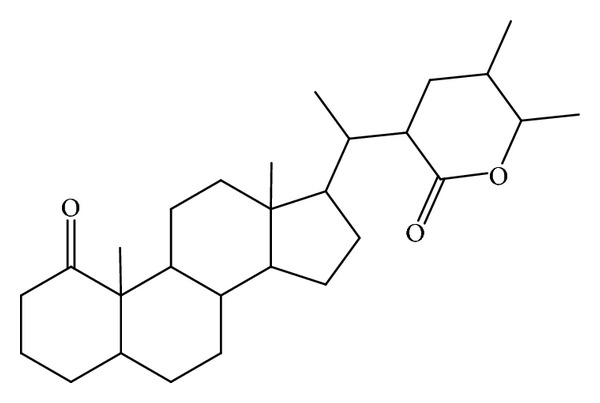
General carbon skeleton of withanolides is shown above and these are known to exert cardiovascular effects.

Is commonly known as Indian cheese maker. A steroidal lactone, withanolide (Scheme 2), isolated from the aqueous extract of fruits of *W. coagulans*, has cardiovascular effect [89]. Alcoholic extract has shown antibacterial and antihelminthic activities [90]. The hot aqueous extract of *W. coagulans* fruits has shown to exert hepatoprotective, anti-inflammatory and antidiabetic effects [91–93]. The hot aqueous extract of *W. coagulans* fruits increases the glucose utilization in isolated rat hemidiaphragm cells [94].

### 4.9. *Cajanus cajan* (Family: Leguminosae)

It is an annual or perennial herb or shrub. It is commonly known as pigeon pea or red gram in English and Arhar in Hindi. It is one of the most important pulse crops cultivated in India [95]. Arhar is consumed in the form of split pulse or dal or, when tender, as a vegetable. The green leaves and tops of the plant are used as fodder and as green manure [96]. *C. cajan *leaves have hyperglycemic activity. LIBS spectra of* C. cajan* clearly reveal that the extract consists of elements like Mg, C, and Ca in the spectral range 200–500 nm. The intensity ratios of Mg/C and Ca/C have been calculated and found that these ratios for *C. cajan* are lower in comparison to the above-mentioned plant extracts known to have antidiabetic activity (Table 2).

### 4.10. *Musa paradisiaca* (Family: Musaceae)

Is a perennial tree-like herb. It is commonly known as banana and is widely found in Northern India. Ayurvedic physicians of Karnataka and Kerala recommended *Musa paradisiaca *for the treatment of urinary stones [97]. The stem juices of *Musa paradisiaca *have been reported for dissolving preformed stones and in preventing the formation of stones in the urinary bladder of rats [98, 99]. The juice of the stem is also used in nervous affectations like epilepsy, hysteria, and also in dysentery and diarrhea. Several sugars comprising fructose, xylose, galactose, glucose, and mannose occur naturally in banana, making it an excellent prebiotic for the selective growth of beneficial bacteria in the intestine [100]. The stem juice of *M. paradisiaca *has hyperglycemic activity [94]. 

Since all the three hyperglycemic extracts are sharing Mg/C as well as Ca/C, their opposite effects on BGL lead us to believe that the ratio of concentration of magnesium and calcium over carbon plays a vital role in determining whether a plant is hyperglycemic or hypoglycemic. Hence, to analyze the role of these elements in diabetes management, understanding of the specific proportion of these elements in all the extracts is essential. Additionally, other essential constituents like H, O, N, and C are found in equal proportion in both the extracts and show no significant role in diabetes management.

## 5. Conclusion 

The present review describes some advanced applications of LIBS. This technique is gaining popularity for qualitative and quantitative analysis of trace elements present in any material. The use of LIBS does not involve complicated sample preparation, is portable and easy to use, with high reproducibility. The technique is based on the principle of the spectral analysis of radiation emanating from microplasma generated by focusing a high power pulsed laser beam on the surface of the sample. The characteristic emission from plasma is recorded as spectrum, which provides a fingerprint of elements present in the target material. Almost all metals and some nonmetals in different matrices have been target elements in trace determinations by LIBS and can be explored further by the pharmaceutical industry. Further research in this direction (detection of glycemic elements from medicinal plants) will be on developing on-site and doing remote analysis of elements, improving precision in measurement, developing accurate calibration procedures and detection limits.

## Figures and Tables

**Table 1:** Intensity ratio of different elements of some antidiabetic plants with respect to C III (229.6 nm).

Intensity ratio (element/ref)	*Cynodon dactylon *	*Emblica officinalis *	*Ficus benghalensis *	*Moringa oleifera *	*Momordica charantia *	*Psidium guajava unripe *	*Trichosanthes dioica *	*Withania coagulans *
C III_229.6 nm_/C III_229.6 nm_	1	1	1	1	1	1	1	1
C_247.8 nm_/C III_229.6 nm_	0.58326	0.87216	0.72842	0.77376	0.99845	0.63213	0.65925	4.43244
Mg II_279.5 nm_/C III_229.6 nm_	1.53617	3.06977	3.19302	1.31796	2.74925	4.26175	1.48591	4.33553
Mg II_280.2 nm_/C III_229.6 nm_	0.70947	2.04378	1.82947	0.84332	2.95627	2.39152	1.13031	3.39804
Ca_393.3 nm_/C III_229.6 nm_	2.58442	39.2093	2.72951	3.37054	1.93864	0.72139	3.38716	1.98661
Ca_396.8 nm_/C III_229.6 nm_	1.95627	18.31737	2.94815	4.18424	1.67391	0.51283	2.02546	0.76354
Ca_422.7 nm_/C III_229.6 nm_	0.98174	5.41176	1.87245	2.74294	0.98351	0.39271	0.55465	

**Table 2:** Intensity ratio of different elements of some hypoglycemic plants with respect to C III (229.6 nm).

Intensity ratio (element/ref)	*C. cajan *	Ripe fruit peel of *P. guajava *	*M. paradisiaca *
C III_229.6 nm_/C III_229.6 nm_	1	1	1
C_247.8 nm_/C III_229.6 nm_	0.64383	0.75183	0.78036
Mg II_279.5 nm_/C III_229.6 nm_	1.77763	1.51083	0.51094
Mg II_280.2 nm_/C III_229.6 nm_	2.10679	0.86273	0.36734
Ca_393.3 nm_/C III_229.6 nm_	2.18003	2.98471	3.39804
Ca_396.8 nm_/C III_229.6 nm_	1.30575	1.57927	1.98661
Ca_422.7 nm_/C III_229.6 nm_	0.57595	0.62948	0.76354
